# A Lecture on Hæmoptysis

**Published:** 1839-01-26

**Authors:** N. Chapman

**Affiliations:** Professor of the Theory and Practice of Physic, in the University of Pennsylvania


### MEDICAL EXAMINER.

DEVOTED TO MEDICINE, SURGERY, AND THE COLLATERAL SCIENCES.

No. 4.] PHILADELPHIA, SATURDAY, JANUARY 26, 1839. [Vol. II.

### A LECTURE ON HÆMOPTYSIS.

*By* N. Chapman, M. D., *Professor of the Theory and Practice of Physic, in the University of Pennsylvania.*

(Reported for this Journal.)

The general principles on which the treatment of hæmorrhage is to be conducted, having now been stated, it will be my next duty to illustrate and enforce them in an application to each individual case.

### HÆMOPTYSIS, OR SPITTING OF BLOOD.

Taking this term in its literal acceptation, it is not at all significant of the pathological state which it is designed to express. As well might a pleurisy be denominated an expectoration of mucus or any other matter, as this affection a spitting up of blood. The act of sputation here is not always performed, the blood, indeed, being more frequently ejected in other and very different modes. Feeling the force of these objections, it has been proposed to entitle this hæmorrhage, *pneumorrhagia.* But this term, as denoting merely a flow or discharge from the lungs, conveys scarcely a more precise or definite meaning.

Numerous divisions of hæmoptysis were formerly made, founded on the difference of causes, or the modes of production. Discarding, however, these varieties, as not belonging to vital hæmorrhage, recent pathologists have a distinction only in reference to the sources whence the blood issues, which are either the mucous, or intercellular tissue of the lungs.

Like all other hæmorrhages, the one of which we are now treating, may be of an active febrile character, or the reverse,—and it is the former that will claim our immediate attention. This is usually ushered in by a sense of weight, sometimes of slight pain or burning in the chest, particularly under the sternum—a dry, hard cough—some shortness and difficulty of breathing—tickling in the larynx, trachea, or bronchiæ—a full, active pulse, and flushed face. But, on other occasions, the symptoms are far more aggravated. Coming on with little premonition, for the most part, though I have seen it preceded for some days by a good deal of pectoral irritation, the attack is characterized by the heaviest oppression, heaving of the chest—diaphragmatic or abdominal breathing—tumid, purple, or livid countenance—distraction of mind—cold skin—dewy perspiration, particularly about the face and neck—feeble, or a full, tumultuous circulation—in the whole, presenting the aspect of the greatest distress, and most imminent danger. Death sometimes takes place in the course of a few minutes, as I once witnessed myself,—and we have heard of an individual who as suddenly died from it in our theatre some short time ago. Thus characterized, it is probably of the nature of pulmonary apoplexy, as it is called.

Cases, too, sometimes occur, introduced more decidedly as fever. There is here a chill, with the pallid, constricted surface—coldness of the extremities—pains in the back and loins—disordered stomach—constipation and lassitude, followed, on reaction, by a full, hard, active pulse, and much heat and excitement. This state may be continued, or betray more or less of an intermittent type. The latter is not uncommon, of which I have seen some instances,—the most remarkable of which was that of a lady, in consultation with Dr. Mitchell, who, for eleven successive days, had hæmoptysis at nine o'clock, precisely, in the morning, always preceded by a slight chill.* Many, with the same type, are recorded, recurring daily, every other day, or fourth day, or weekly, or monthly,—thus conforming strictly to the law of periodicity, observed by intermittent fever.

As an example of the quotidian form, Reil mentions the case of a woman, who had an attack of hæmoptysis every morning for two years,—and Thompson furnishes an instance of a tertian, that regularly returned for more than a year,—Bursenius, one of a quartan, that reverted with great exactness for a long time,—and Richter saw another, where the hæmorrhage was repeated monthly for twenty-five years. Tulpius, indeed, relates cases of thirty, and even forty years' continuance of this type. But here, the catamenia were suppressed. Lusitanus, Shenk, Meyer, and Mead, have reported similar facts, without, however, noticing the state of the menstrual function,—and Blanchard had a case reverting every three months, from an interruption of hæmorrhois. An attack of hæmoptysis is very apt to be followed by others, though comparatively seldom with any periodical precision.

In the mode, as well as in the quantity of blood discharged, there is a difference. It sometimes comes up, attended by rattling in the windpipe, as if the tube were stuffed with phlegm, and by an irresistible propensity to cough,—which state of things may terminate at once, with a discharge of bloody sputa only, or of a single mouthful of pure blood, or endure for a few days or for a longer period. There are instances, probably, of pulmonary apoplexy, in which it issues so copiously as to appear like a stream from the mouth. Two quarts, at least, I once saw come away in twenty or thirty minutes. Laennec says, that he has known thirty pounds lost in about fifteen days,—and, in a very extraordinary case, ten pounds in forty-eight hours.

* The wife of the Rev. Mr. Sandford.

When so profuse, a sort of convulsive elevation of the diaphragm takes place, as in puking, which has probably led to the common expression, of vomiting of blood, in this affection.

The causes, generally, of hæmorrhage, may occasion hæmoptysis,—though there are some which more particularly conduce to this event.

1st. It is well ascertained that a predisposition to it, is laid in a certain conformation or structure. Thus, a narrow thorax, and prominent shoulders, a long neck, with a delicate make, and sanguine temperament, seem particularly to invite such attacks. Connected, or otherwise, with such a configuration and temperament, this predisposition is often transmitted as an inheritance, descending directly, or through intermediate generations, to the whole, or greater part of a numerous family. On what it depends, is not always apparent,—though a strumous or tubercular diathesis is mostly betrayed, or may be suspected.

2d. The period of life is to be deemed a second cause of predisposition. Hæmoptysis rarely happens earlier than the twelfth, and is not common after the thirty-fifth year,—chiefly prevailing between the ages of fifteen and twenty-five, and especially at the season of puberty. Cullen supposes this to be owing to a want of due balance in the aortic and pulmonary circulations, from the continuance of the growth and expansion of the thorax, after the other portions of the body are completed, by which blood is unequally determined to the lungs.

It may be, in part, at least, on this account, that in females, in whom the change is more conspicuous at the period of maturity, are more liable to such attack, than males, though it appears that at all times they have rather a greater susceptibility to this hæmorrhage.

3d. That tubercles of the lungs predisposes to hæmoptysis is indisputable. Nor, perhaps, is it less true that it is occasioned by other derangements of these organs, or of the heart, liver, or spleen, interrupting the freedom of circulation, as well, also, as by gastric and intestinal irritation reflected on the lungs.

4th. Certain classes of people are prone to hæmoptysis, from their avocations or habits, among which are those who work in a bent position, as tailors and shoemakers, as well as such as are exposed in their operations to acrid or otherwise irritating inhalations.

The late Professor Rush tells us, that those religious denominations who do not sing, and mostly worship silently, are very subject to it, from weakness of lungs, owing to the want, in this mode, of adequate exercise of these organs. My own experience, however, does not confirm this observation. Living in the "City of Friends," I have seen no peculiar liability, in this description of people, to be thus affected. Clergymen, on the contrary, are exceedingly exposed to hæmoptysis, which has been ascribed, though I think erroneously, to the performance of the services of the pulpit. That it cannot be so, to the extent averred, is shown by the comparative immunity of lawyers, legislators, and lecturers, each of whom, harangue more constantly and loudly than preachers. May it not be referred to individuals with frail constitutions, and strong tendencies to pulmonary disorders, under the solemn impresssion of the uncertainty of life assuming the sacred office? Be this as it may, the fact appears to me unquestionable, that this hæmorrhage is far more frequently to be witnessed in connexion with an undue exertion of the lungs, than the reverse, or the comparatively quiescent state, to which an allusion has been made.

As regards public singers, especially those of the opera, where the vocal powers are strained to the utmost, it is acknowledged that they are singularly liable to hæmoptysis, or if they escape it, they soon begin to suffer from some pulmonary affection, and either prematurely die, or retire from their profession, with a shattered voice and infirm health. Three or four years, I was informed by one of them, are, perhaps, the average of the full preservation of their powers.

5th. Nor are climate and particular localities without an influence in the production of this hæmorrhage, it being most prevalent in the medium latitudes, where the weather is cool, damp, and austere, especially along the sea coast, and more in elevated or mountainous, than in flat districts of country.

Congenital or acquired predispositions are excited into action by a variety of circumstances, or the latter may produce the same effect without any such tendencies. Not the least common of these, besides long or loud speaking or singing, already referred to, are sudden bursts of laughter—paroxysms of anger, or other violent mental emotions—great exertions, especially raising heavy weights—irregular habits of living—the suppression of some customary discharge, as hæmorrhois, epistaxis, or the catamenia—as well as the healing of old ulcers, or the sudden cure or repulsion of cutaneous eruptions—the metastasis of gout or rheumatism—an exposure to a heated and impure or very rarified atmosphere—and above all, the vicissitudes of weather, producing catarrhal or other pectoral affections. I have frequently seen it proceeding from the irritation of an elongated uvula, and sometimes, though rarely, from enlarged tonsils. Cases, too, have been reported, where an attack has followed the tying up of large arteries in surgical operations, so as to throw on the lungs an oppressive quantity of blood; and the loss of a limb, in the same way, more constantly induces it.

Nevertheless, though hæmoptysis is excited by the causes enumerated, it is still true, however extraordinary it may appear, that it occurs more frequently at night, and in sleep, when, of course, there is the least corporeal or mental agitation. Of this, at least, I am persuaded, that of the cases I have seen, a majority took place, under the circumstances mentioned.

Whether it is to be imputed to an increased susceptibility, acquired by the state of repose, as has been alleged, I shall not take upon myself positively to determine. It is altogether a curious fact, and has never, perhaps, been very satisfactorily elucidated, or explained. But I cannot help suspecting that it is referrible to the horizontal posture, and more particularly to the bending of the lower extremities in sleep, which, unquestionably, has great influence in determining blood to the lungs.

(To be continued.)

